# Association of Maternal Feeding Style with Fruit and Vegetable Consumption in Saudi Preschoolers: A Nationwide Cross-Sectional Study

**DOI:** 10.3390/nu15224735

**Published:** 2023-11-09

**Authors:** Amal Abdulaziz Al-buobayd, Hala Hazam Al-Otaibi, Hoda A. S. Farag

**Affiliations:** Department of Food and Nutrition Science, College of Agricultural and Food Science, King Faisal University, Al-Ahsa 31982, Saudi Arabia; 220001585@student.kfu.edu.sa (A.A.A.-b.); hfarag@kfu.edu.sa (H.A.S.F.)

**Keywords:** fruit, vegetable, preschool children, parental feeding style, Parental Feeding Style Questionnaire, encouragement feeding

## Abstract

Parental feeding style (PFS) remarkably influences fruit and vegetable (F&V) consumption in preschoolers. This study aimed to determine the association between PFS and preschoolers’ F&V consumption, as influenced by socioeconomic factors. A nationwide cross-sectional study was conducted among 1418 mothers of children aged 3–5 years in Saudi Arabia. Multinomial logistic regression model analysis was performed to assess the association between PFS and children’s daily F&V intake using the Parental Feeding Style Questionnaire. The influence of socioeconomic factors on this association was also evaluated. For mothers with university degrees, encouragement, emotional, and instrumental feeding enhanced their children’s intake of F&Vs, fruits, and vegetables, respectively. Children from families earning SAR >10,000 monthly had increased F&V intake with encouragement feeding and increased fruit intake with emotional feeding. However, F&V intake was reduced in children of working mothers with controlling feeding styles. Breastfeeding for <6 months was associated with increased F&V intake through emotional feeding. The most prevalent feeding style was encouragement, followed by emotional, with control and instrumental styles being less common. This study provides strong evidence on the association between PFS and daily F&V intake in Saudi preschoolers. Interventional and longitudinal studies on PFS are required to confirm these findings.

## 1. Introduction

Healthy living in people of all ages is required to meet the sustainable development goals (SDGs). Significant progress has been made in increasing life expectancy and decreasing maternal and child mortality [1]. However, more research is required to promote healthy habits, such as consumption of a balanced diet rich in fruits and vegetables (F&Vs). According to the World Health Organization (WHO), adults and children should consume 400 g (five servings) of F&Vs daily [2]. Insufficient intake of F&Vs is a major risk factor for chronic diseases, accounting for 14%, 11%, and 9% of mortalities from gastrointestinal cancer, heart disease, and stroke, respectively, worldwide [3,4].

The consumption of F&Vs is considered a vital part of nutrition at all stages of life. According to a 2015 UNICEF report, children under the age of 5 years are considered a valuable resource worldwide, and focusing on this age group will help accomplish the SDGs by 2030 [5]. The preschool age (3–5 years) is crucial for physical and mental development [6]. Consuming F&Vs during childhood has a positive impact on long-term health outcomes, can aid in weight control, and lowers the risk of childhood obesity, which is increasing globally [7].

However, the recommended daily intake of F&Vs for children varies between countries and health organizations. Few countries have specific dietary recommendations, and some European countries (Norway, the Netherlands, Greece, Finland, and Denmark) have specific dietary guidelines ranging from 100 to 500 g or one to three servings daily [8]. The United States recommends that children aged 4–8 years should consume 1–1.5 cup of fruits and 1.5 cup of vegetables daily [6].

Other nations, notably the United Kingdom, have accepted the WHO recommendation of at least 400 g per day for the general population, including children [9,10]. The recommended daily intake of F&Vs for children aged 4–8 years in the Kingdom of Saudi Arabia (KSA) is five servings per day [11]. These discrepancies are most likely due to local factors, such as dietary traditions and available resources. Nonetheless, there is agreement that consuming F&Vs daily is essential for good health [4,12].

Previous research found that a considerable proportion of children aged 3–5 years do not meet their daily F&V needs. In England, for example, only 17% of boys and 19% of girls consume the recommended five or more servings of F&Vs per day [13]. According to a study conducted in Hunan, China, 35.6% and 42.4% of children aged 4–5 years consume vegetables and fruits more than once per day, respectively [14]. A Lebanese study found that only 17.8% of children consumed the recommended daily intake of vegetables, and only 34.3% consumed fruits daily [15]. In Oman, 29% and 34% of children aged 2–5 years were found to consume vegetables and fruits two or more times daily, respectively [16]. Similarly, in a study in the KSA, only 42% of preschool children were found to consume fruits daily [17].

It should be noted that mothers’ dietary habits and behaviors play a crucial role in shaping their children’s eating habits through role modeling. Several studies have found that children’s food preferences are influenced by their mothers’ dietary habits and F&V consumption. Groele et al. [18] found a correlation between the daily servings consumed by mothers and those consumed by their children in Poland and Romania. Another study in Oman by Almaamary et al. [16] found that mothers who do not like F&Vs may influence their children’s preferences. Robson et al. [19] reported the same finding.

The association between high family socioeconomic status, especially parental educational level and family income, and children’s F&V consumption has been well documented [20,21]. The educational level of mothers plays a significant role in shaping the F&V intake of preschool children. Mothers with higher education tend to have a better understanding of nutrition, leading to healthier family food choices. Their awareness of the nutritional value of F&Vs enables them to encourage their children to consume these foods. This maternal education serves as a crucial factor in promoting healthy F&V consumption patterns during early childhood, as evidenced in studies from Finland, France, and South Korea [22,23,24].

High-income households are more likely to purchase healthy foods that are rich in fiber and low in salt and added sugar, indicating a healthy-eating home environment [25,26]. Moreover, the bond built between a mother and her infant through breastfeeding is critical in determining a child’s future satiety responsiveness later in life. A mother’s continuous consumption of F&Vs can also influence the development of a child’s food choices. This is because children develop a taste for the flavors that they are exposed to during breastfeeding [27,28].

Parental feeding style (PFS) influences children’s food intake, especially that of F&Vs [29,30]. The ways parents interact with their children during mealtimes play a significant role in the development of healthy eating practices and food preferences. These styles include “control”, which involves setting a specific time for the child to have a snack; “encouragement”, which involves praising the child if they finish the food provided; “emotional”, which involves rewarding a child with food when they behave well; and “instrumental”, which involves giving the child food when they feel bored to make them feel better [29,30,31].

Studies have shown that parents who adopt a healthy and balanced feeding style help their children develop healthy eating habits, which can help prevent nutrition-related diseases and promote overall health and growth [32,33]. A study conducted in the Netherlands used the Parental Feeding Style Questionnaire (PFSQ) scale to measure the feeding style of parents in relation to F&V and sugar-sweetened beverage (SSB) consumption. The children of parents who used encouragement and control feeding styles consumed more F&Vs and fewer SSBs. The study suggested that the encouragement feeding style helps children learn to enjoy the taste of healthy foods [32]. A study conducted in Turkey on children aged 3–5 years reported that encouragement and instrumental feeding styles led to greater dietary diversity in children [33]. Additionally, a study conducted in Sri Lanka found that parents of preschool children were more likely to use control and encouragement feeding styles [34].

Previous studies conducted in the KSA have focused on the impact of parental beliefs, attitudes, and practices regarding child feeding on preschool-aged children and mothers’ concerns about their children’s weight or consumption of unhealthy food. Common scales used among Saudi preschool children are the Child Feeding Questionnaire and the Children’s Eating Behavior Questionnaire [35,36,37]. However, the PFSQ has never been used to assess PFS regarding daily F&V intake in this age group. Owing to the scarcity of research in this area, there is an urgent need for studies on the relationship between PFS and F&V consumption among children aged 3–5 years in the KSA. The specific research questions addressed in this study are as follows:

- What is the average daily consumption of F&Vs among preschool-aged children in the KSA?
- What are the different feeding styles associated with F&V consumption among preschool-aged children in the KSA?
- How does the interaction between feeding styles and socioeconomic factors influence F&V consumption?

## 2. Materials and Methods

### 2.1. Design and Sample Study

This cross-sectional study was performed to collect data from the Northern, Southern, Central, Eastern, and Western administrative regions of Saudi Arabia that were recorded from 2 July 2022 to 29 November 2022 through an electronic questionnaire administered via the Internet using Google Forms. According to data from the General Authority for Statistics (GAStat) in Saudi Arabia, there are 2,844,501 children aged 0–5 years [38]. The sample size was calculated based on an expected correlation coefficient of 0.15, a strength of 80%, and an alpha level of 0.05, and it was found that the number of participants had to be at least 385 [39].

We employed a non-probability convenience sampling method in the virtual environment, specifically using a snowball-type recruitment approach, for data collection [40]. Data on children were collected from their mothers, as mothers play a significant role in child feeding in many countries [35,41]. The survey link included the objectives and procedures of the study, the privacy policies, and an explanation of participation. Mothers who were selected and agreed to participate were instructed to provide data on only one child. Survey links were distributed on four social media platforms to ensure a high rate of response [42] (WhatsApp, Telegram, Instagram, and Twitter) from all over the KSA, from 2 July 2022 to 29 November 2022.

The researchers encountered difficulty in directly engaging with mothers of kindergarten students across all regions in Saudi Arabia. This posed challenges in identifying suitable participants. Consequently, they had to resort to utilizing social media platforms to locate and recruit the participants. Previous research findings have demonstrated that utilizing social media for sampling is an effective and efficient method to recruit study participants. This approach enables a larger sample size, a shorter completion time, and reduced application costs [40,43].

Participation in the study was voluntary, with 1682 participants. The criteria for joining the study were determined, including mothers of Saudi children between the ages of 3 and 5 years old who did not have any obvious health problems and who completed the questionnaire.

This was decided to ensure that any impact on children’s feeding habits could be attributed to factors other than underlying health issues. Non-Saudi mothers and children who did not provide their informed consent to participate, who had missing data in the questionnaire, and who did not meet the health criteria and other conditions specified for participation were excluded. With the aim of ensuring the validity and the reliability of the results and focusing on the impact of factors associated with children and their families, as well as the ability of researchers to extract accurate and reliable data, data were collected from 1418 mother–child pairs included in the final analysis. Among them, 264 pairs (15.6%) with incomplete participation were excluded to enhance the precision of the estimates, while missing data introduced significant variability.

### 2.2. Independent Variables

#### 2.2.1. Demographics and Other Potential Confounders

Data on the mother’s age, marital status, employment status, education level, family monthly income, and region of residence; the father’s age, education level, and employment status; and the child’s age, sex, number of siblings, kindergarten attendance, and breastfeeding duration were collected. These variables were selected based on previous studies that have shown their impact on children’s F&V intake [18,25,32,44,45].

#### 2.2.2. Mothers’ Daily Intake of F&Vs

F&V consumption was assessed based on the responses to the question “How many servings of F&Vs do you usually eat daily?”, which has been validated in previous studies [46,47]. The study defined one serving as one cup of raw vegetables, half a cup of cooked vegetables, or one medium-sized fruit according to the recommended daily amount of F&Vs for Saudi adults aged 19–50 years, with seven to eight servings daily [11]. The F&V consumption responses were categorized into none, one serving, two servings, three to four servings, five to six servings, and seven servings or more.

#### 2.2.3. PFS

PFS was assessed using the PFSQ, a 27-item questionnaire developed by Wardle et al. [48] in English, and it has been translated into many languages [32,49,50,51]. The PFSQ was designed for use in a study examining obesity risk in children of mothers with obesity and those with normal weight.

The scale has four subdimensions: emotional feeding (five items, e.g., “rewarding the child with food when they behave well”), instrumental feeding (four items, e.g., “giving the child food when they feel bored to make them feel better”), encouragement feeding (eight items, e.g., “praising the child if they finish the food provided”), and control feeding (ten items, e.g., “setting a specific time for the child to have a snack”). Respondents rated each item on a 5-point Likert scale (ranging from “never” to “always”). A translation and back-translation procedure was used to create an Arabic version, and the completed questionnaires were pretested with a convenience sample of 10 mothers. Cronbach’s alpha coefficients varied among the feeding styles: emotional (0.924), instrumental (0.916), encouragement (0.838), and control (0.857). The obtained values indicated excellent to good internal consistency, which is similar to the findings of studies conducted in several countries [32,49,50].

### 2.3. Dependent Variables

#### F&V Intake in Children

Young children are sometimes in the care of others, such as grandparents or paid babysitters, during the day while their parents are at work. During these times, parents’ ability to accurately report their children’s dietary intake, especially that of F&Vs, is influenced by other elements, such as memory and the time required by the assessment method used [52]. In addition, the online nature of this study required a valid and reliable short assessment method that was easy to complete and recall. To assess children’s daily F&V intake, mothers were asked to answer two questions: “how many servings per day they consumed fruit?; how many servings per day they consumed vegetables?”. To help mothers provide accurate information about their children’s food intake, photographs of F&V portion sizes were included. All responses were coded based on the reported number of serving spoons of vegetables (one serving spoon of vegetables = 50 g) or pieces of fruit (one pieces of fruit = 100 g). The response options for vegetable consumption were as follows: “less than one serving spoon = 0.5 serving spoon/day”, “one to two serving spoons = 1.5 serving spoon/day”, “three to four serving spoons = 3.5 serving spoons/day”, “five to six serving spoons = 5.5 serving spoons/day”, and “more than six serving spoons = 7 serving spoons/day”. The response options for fruit consumption were as follows: “less than one piece = 0.5 piece/day”, “one piece = 1 piece/day”, “two pieces = 2 pieces/day”, and “more than two pieces = 3 pieces/day”, similar to the guidelines used in previous studies [32,53,54]. Children who consumed, on average, 1.5 pieces/day of fruit and 3 serving spoons/day of vegetables were categorized as meeting the daily requirements of F&V intake.

### 2.4. Statistical Analysis

Descriptive analyses were performed, and data are presented as frequencies, percentages, means, and standard deviations. Continuous data were analyzed using the independent-samples *t*-test and categorical variables using the chi-square test, followed by multinomial logistic regression analysis to examine the relationship between PFS and the children’s F&V intake, as influenced by other potential factors such as socioeconomic variables. All analyses were carried out using IBM SPSS Statistics version 29 software, with the significance level set at *p* < 0.05.

## 3. Results

Table 1 shows the characteristics of the 1418 participants and the differences in fruit and vegetable intake across these characteristics. In total, 35.7% of the children consumed ≥1.5 pieces/day of fruits, while 59.3% consumed ≥3 serving spoons/day of vegetables. There were more girls (65.2%) than boys among the children included, and the average age of the children was 4.18 ± 0.83 years, with 43.7% having two or more siblings. Almost half of the children were attending kindergarten (47.1%), with no significant difference between them. A total of 61% of the mothers in this study breastfed their children for ≥6 months, and 58.3% and 65.4% of the children consumed the recommended daily intake of F&Vs, respectively, with a significant difference. The majority of children from the Western region (27.8%) consumed ≥3 serving spoons/day of vegetables (32.1%), and the majority of those from the Eastern region (26.6%) consumed ≥1.5 pieces/day of fruits (31.6%), with significant differences between the regions. The mean age of the mothers was 32.19 ± 6.18 years, and that of fathers was 36.81 ± 7.37 years. Most mothers (88.2%) and fathers (78.8%) were married and had high education levels. However, 57.4% of the mothers were unemployed. Approximately half of the families had a monthly income of SAR > 10,000 (Saudi Arabian riyals). Regarding the mothers’ consumption of F&Vs, 22.4% consumed ≥7 servings per day, with a significant difference between the variables.

### 3.1. PFSs and Children’s Consumption of F&Vs

Table 2 shows the results of the independent-samples *t*-test indicating statistically significant differences between PFSs and F&V consumption.

### 3.2. Association of PFS and Children’s Consumption of F&Vs with Demographic Factors

Only variables with significant differences were included in the multinomial logistic regression analysis (Table 3 and Table 4). Children of employed mothers who used control feeding had low intakes of F&Vs (odds ratio (OR) = 1.016; 95% confidence interval (CI) 0.989–1.043; *p* ≤ 0.001 and OR = 1.013; 95% CI 0.980–1.046; *p* ≤ 0.05, respectively). Mothers with a higher education level used emotional (OR = 0.936; 95% CI 0.877–0.999; *p* ≤ 0.05) and encouragement (OR = 1.067; 95% CI 1.023–1.112; *p* ≤ 0.05) feeding to increase fruit intake in their children, whereas they used instrumental (OR = 1.07; 95% CI 1.002–1.143; *p* ≤ 0.05) and encouragement (OR = 1.062; 95% CI 1.029–1.096; *p* ≤ 0.001) feeding to increase vegetable intake. In contrast, mothers with family incomes SAR >10,000 who used emotional and encouragement feeding had children who consumed more F&Vs (OR = 1.095; 95% CI 1.042–1.51; *p* < 0.001 and OR = 1.049; 95% CI 1.015–1.085; *p* ≤ 0.05, respectively). A higher family income influenced the association between encouragement feeding and intake of ≥3 serving spoons/day of vegetables (OR = 1.031; 95% CI 1.006–1.057; *p* ≤ 0.05).

In addition, longer breastfeeding duration influenced the association between emotional feeding and increased intake of F&Vs (OR = 1.051; 95% CI 1.016–1.88; *p* ≤ 0.05 and OR = 1.070; 95% CI 1.039–1.101; *p* ≤ 0.001, respectively). Finally, fruit (OR =1.113; 95% CI 1.041–1. 190; *p* ≤ 0.05) and vegetable (OR = 1.049; 95% CI 1.022–1.077; *p* ≤ 0.001) consumption increased among children whose mothers consumed ≥7 serving/day of F&Vs and used encouragement feeding.

## 4. Discussion

This nationwide study was conducted in the KSA to provide a comprehensive understanding of the relationship between PFS and F&V consumption among children aged 3–5 years, as influenced by socioeconomic factors, such as mother’s education level, employment status, mother’s daily F&V consumption, household income, and duration of breastfeeding. Approximately 60% of the children consumed ≥3 serving spoons/day of vegetables compared with a low prevalence of fruit consumption (35.7%; ≥1.5 pieces/d). These findings are not completely consistent with those from previous national and international studies that have shown a reduction in F&V consumption among children [16,17,55]. Our finding that approximately 66% of children who were breastfed for >6 months consumed ≥3 serving spoons/day of vegetables suggests that breastfeeding may help shape a child’s taste preferences [28]. Additionally, more than half of the children attended kindergarten, where vegetables are frequently more easily available than fruits; for example, vegetables are frequently offered at meals or as snacks at kindergarten, whereas fruits are usually regarded as snacks [52]. Another explanation is that most children with two or more siblings consumed adequate amounts of F&Vs (45.1% and 44.5%, respectively). Finally, children may be more familiar with cooked vegetables served regularly at home than with fresh fruits [56]. Siblings can influence each other’s behavior; for example, if an older sibling follows a good diet, the younger sibling is more likely to follow suit [45].

More than two-thirds of the mothers (77.6%) did not meet their daily F&V requirements, similar to the findings of previous studies among Saudi women [46,47]. To set a good example for their children, mothers must actively include F&Vs in their diets [18,19]. Mothers can considerably influence their children’s eating habits by adopting healthy practices, filling their homes with nutritious food, involving their children in cooking activities, and supporting balanced family meals.

Significant differences were observed between the four sub-dimensions of PFS regarding children’s daily F&V intake. It was found that the use of encouragement feeding was the highest, followed by emotional feeding, control feeding, and instrumental feeding. These findings are consistent with those of previous studies conducted in Sri Lanka [34] and Indonesia [57], where the most-used style was encouragement feeding. In contrast, other studies have reported control feeding to be the most commonly used style [32,44]. These differences can be explained by the cultural and social factors that influence parental feeding practices [58,59,60].

Many Saudi women now work outside their homes to support their families. However, mothers are not relieved of their usual household duties but are instead burdened with both their job and household responsibilities. The current study found that the use of control feeding by working mothers was associated with decreased fruit intake. A similar study conducted to examine the association of maternal parenting styles with healthy and unhealthy food intake in Australian preschool children found statistically significant interactions indicating that mothers’ employment status may moderate the relationship between parenting style (authoritarian and permissive) and low fruit intake (*p* = 0.019), increased SSB consumption (*p* < 0.001), and consumption of hot takeaway food (*p* = 0.048) in children [56]. Further research is needed to examine the influence of maternal employment status, with more focus on the impact of other potential factors, such as working hours and work pressures, which may provide further insights into the association between PFS and children’s F&V intake.

Education is of the utmost importance in enlightening minds, promoting continuous learning, understanding relationships, and guiding personal matters. Education effectively contributes to the acquisition of new knowledge and serves as a powerful tool for self-development and family progress. The findings of the present study indicate that encouragement feeding tends to promote high F&V intake in children whose mothers had a university degree or higher, consistent with the findings of previous studies [32,50,61]. Furthermore, mothers with high education levels used instrumental feeding only to increase vegetable intake in their children, which could be due to parents using vegetables to comfort or reward their children when they are bored. The combination of these two feeding styles may help children meet their daily F&V intake requirements [62]. Emotional feeding was significantly associated with higher fruit consumption in children in the present study. Contrastingly, Lo et al. [50] found that control feeding was the most common style used by highly educated parents to increase F&V intake in preschool children in Hong Kong. In general, educated mothers are more likely to promote healthy behaviors in their children, and a study conducted in Norway revealed that children whose mothers completed university education consumed an average of 79.7 g of vegetables daily, compared with an average of 28.9 g in children whose mothers had a lower education level [63]. Other studies confirmed these results [18,62].

Supporting and encouraging mothers to continue breastfeeding and feeding their children healthy foods is essential. Previous studies [64,65,66] have suggested that mothers need continuity, support, and encouragement to achieve this. According to previous studies, a longer duration of breastfeeding was positively associated with a higher amount of F&V consumption and a greater diversity of vegetables consumed by children and adults. The flavors of food consumed by mothers are likely to be passed on to their children via breast milk [28,67,68,69]. However, mothers who breastfed their children for >6 months were more likely to use emotional feeding to increase F&V intake. To the best of our knowledge, no previous study has investigated the association between PFS and F&V intake, as influenced by breastfeeding among preschool children. There are several possible explanations for this association. First, breastfeeding mothers may be more in tune with their babies’ needs because they are more closely connected to them physically and emotionally. Second, breastfeeding mothers are more likely to have a positive view of their babies’ ability to self-regulate their eating habits.

Several studies have investigated the relationship between preschool children’s F&V intake and their family’s socioeconomic status and found that children from high-income families often have better access to fresh F&Vs, as their families can afford to buy a wide range of fresh vegetables [25,26]. This greater availability can have a favorable impact on a child’s F&V consumption. However, in the present study, encouragement feeding was associated with increased F&V consumption when the monthly income was more than SAR 10,000, whereas the emotional feeding style increased fruit intake only. This is consistent with the findings of studies conducted by Lim et al. [26] and Mackenbach et al. [70], who indicated that children from high-income households were more likely to consume two servings of F&Vs/day (OR = 0.29, *p* = 0.001), while low-income individuals consumed fewer F&Vs daily. Nevertheless, higher income is not always associated with nutritious food intake. Raaijmakers et al. [71] reported a contrasting association between the instrumental feeding style and increased consumption of high-calorie foods such as candy (66.2%) and lower intake of healthy foods among high- and middle-income children.

Children who grew up with parents who consumed F&Vs were more likely to consume the required daily servings. One approach for increasing children’s consumption of F&Vs is healthy parental role modeling [25,63,72].

Encouragement feeding was associated with increased daily consumption of F&Vs in children whose mothers met their daily requirements of F&V intake, encouraging children to follow a healthy diet by offering a variety of nutritious foods that stimulate their appetite and lead to an overall increase in their food consumption [25]. Increased consumption has also been observed in children who are frequently exposed to healthy foods, contributing to their acceptance of these foods [63,73].

This study has some limitations. First, the study design was cross-sectional; therefore, a causal relationship could not be established between the results. Second, a self-administered questionnaire was completed by the mothers, which exposes them to under-reporting F&V intake; there might be some situations where mothers inaccurately estimated their children’s F&V consumption, such as when their children were in kindergarten or when they were at work. Third, findings from participants with higher education levels and household incomes might not represent the broader population accurately; thus, more studies need to be conducted among diverse social backgrounds. However, this does not diminish some important strengths of our study. This unique nationwide study included mothers from all regions of the KSA. The sample size was large, which increased the accuracy of the results and reduced bias. A well-adapted standardized instrument was used to assess PFS, thereby enhancing the accuracy of the data and the reliability of the results. Actual F&V consumption was measured using a previously validated and reliable method, thereby significantly reducing over- or under-estimation, which could have resulted from recall and self-reports. The age group studied is one of the strengths of this study, as preschool age is an essential developmental stage for adopting healthy eating habits. Using an electronic questionnaire allowed the participants to answer comfortably and honestly without feeling embarrassed. Furthermore, this study linked the data of both mothers and children, which was an additional strength of the study. Finally, the findings of this study could shape upcoming government policies and initiatives aimed at enhancing children’s health.

## 5. Conclusions

The consumption of F&Vs among preschool children is almost satisfactory, indicating a healthy nutritional intake that reduces the risk of future chronic diseases. This aligns with the third goal of the SDGs, “Good Health and Well-being”, which the KSA aims to achieve for all residents by 2030. This study expands our understanding of the effect of PFS on F&V intake among preschoolers. Also explored were the impact of maternal education level, maternal employment status, family monthly income, breastfeeding duration, and maternal daily F&V intake on the association between PFS and children’s F&V intake.

The results indicated that the most common feeding styles among Saudi mothers to increase F&V intake in preschool children included encouragement and emotional feeding, while the less commonly used styles included control and instrumental feeding.

The observations in this study are unique, highlighting significant gaps in knowledge that require additional investigation. Therefore, we recommend the following:

1. In recent times, a series of social developments have been observed with the entry of more women into the labor market in the KSA, causing other family members to be involved in the care of children, such as fathers and grandparents. Therefore, the role of fathers and grandparents in influencing children’s eating behaviors within the Saudi family context warrants further investigation.
2. It has been reported that women use the encouragement feeding style, while men use the control feeding style; however, there have been shifts over time, demonstrating that parents might adapt their interactions to their children’s growth [74]. Longitudinal research is required to investigate these changes in children’s eating behaviors.
3. Saudi mothers traditionally use food as a reward to encourage desirable behaviors, i.e., emotional feeding [75]. However, in the present study, the primary PFS used to increase F&V intake in preschool children was the encouragement feeding style, especially among mothers with higher education levels and incomes. Therefore, there is a need to investigate the relationship between PFS and other socioeconomic classes to gain a more comprehensive understanding.
4. The eating patterns of preschoolers can differ substantially due to various factors, such as individual food preferences, cultural influences, family habits, socioeconomic status, and exposure to different foods and eating environments, and it is unclear whether they typically eat more fruit or more vegetables daily [56,63]. The current study observed a low daily fruit intake in preschool children. Therefore, more research on personal tastes, family food traditions, and cultural aspects is required to understand their roles in fruit consumption.
5. The study findings provide a foundation for implementing practical measures in the future. These insights enable the establishment of effective strategies, ensuring a comprehensive approach to address challenges and promote healthier lifestyles. By leveraging this understanding, concrete actions can be taken to facilitate positive dietary changes effectively.

## Figures and Tables

**Table 1:** Participants’ characteristics and differences in fruit and vegetable intake across these characteristics (*n* = 1418).

Variable	All Participants	Fruits χ^2^/t		*p* Value	Vegetables χ^2^/t		*p* Value
		<1.5 Pieces/d 912 (64.3)	≥1.5 Pieces/d 506 (35.7%)		<3 Serving Spoons/d 577 (40.7%)	≥3 Serving Spoons/d 841 (59.3%)	
		Mean (SD) or *n* (%)			Mean (SD) or *n* (%)		
Children							
Age (years)	4.18 ± 0.83	4.15 ± 0.84	4.13 ± 0.82	0.951	4.11 ± 0.84	4.2 ± 0.82	0.0922
Sex							
Male	493 (34.8)	290 (31.8)	203 (40.1)	0.051	197 (34.1)	296 (35.2)	0.682
Female	925 (65.2)	622 (68.2)	303 (59.9)		380 (65.9)	545 (64.8)	
Number of siblings							
None	363 (25.6)	223 (24.5)	140 (27.7)	0.791	153 (26.5)	210 (25)	0.432
One	436 (30.7)	298 (32.7)	138 (27.3)		179 (31)	257 (30.6)	
Two or more	619 (43.7)	391 (42.9)	228 (45.1)		245 (42.5)	374 (44.5)	
Attending kindergarten							
Yes	668 (47.1)	454 (49.8)	412 (42.3)	0.136	257 (44.5)	411 (48.9)	0.109
No	750 (52.9)	458 (50.2)	292 (57.7)		320 (55.5)	430 (51.1)	
Breastfeeding duration							
Less than 6 months	544 (38.4)	333 (36.5)	211 (41.7)	0.031 *	253 (43.8)	291 (34.6)	0.000 ***
6 months or more	874 (61.6)	579 (63.5)	259 (58.3)		324 (56.2)	550 (65.4)	
Parents							
Mother’s age (years)	32.19 ± 6.18	31.96 + 6.12	32.62 + 6.26	0.54	31.84 ± 6.23	32.43 ± 6.12	0.075
Mother’s marital status							
Widow	45 (3.2)	23 (2.5)	22 (4.3)	0.171	14 (2.4)	31 (3.7)	0.305
Divorced	122 (8.6)	79 (8.7)	43 (8.5)		46 (8)	76 (9)	
Married	1251 (88.2)	810 (88.8)	441 (87.2)		517 (89.6)	743 (87.3)	
Mother’s education level							
Secondary school or lower	300 (21.2)	211 (23.1)	89 (17.6)	0.008 *	140 (24.3)	160 (19)	0.018 *
University or higher	1118 (78.8)	7,1 (76.9)	417 (82.4)		437 (75.7)	681 (81)	
Mother’s employment status							
Unemployed	814 (57.4)	535 (58.7)	279 (55.1)	0.043 *	348 (60.3)	466 (55.4)	0.047 *
Employed	604 (42.6)	377 (41.3)	227 (44.9)		229 (39.7)	375 (44.6)	
Region of residence							
Eastern	377 (26.6)	217 (23.8)	160 (31.6)	0.000 ***	162 (28.1)	215 (25.6)	0.000 ***
Central	254 (17.9)	183 (20.1)	71 (14)		126 (21.8)	128 (15.2)	
Western	394 (27.8)	273 (29.9)	121 (23.9)		124 (21.5)	270 (32.1)	
Northern	230 (16.2)	133 (14.6)	97 (19.2)		77 (13.3)	153 (18.2)	
Southern	163 (11.5)	106 (11.6)	57 (11.3)		88 (15.3)	75 (8.9)	
Father’s age (years)	36.81 ± 7.37	36.4 ± 7.03	37.55 ± 7.91	0.055	36.32 ± 7.79	37.15 ± 7.05	0.054
Father’s educational level							
Secondary school or lower	332 (23.4)	218 (23.9)	114 (22.5)	0.558	161 (27.9)	171 (20.3)	0.101
University or higher	1086 (76.6)	694 (76.1)	329 (77.7)		416 (72.1)	670 (79.7)	
Father’s employment status							
Self-employment	181 (12.8)	99 (10.9)	82 (16.2)	0.054	57 (9.9)	124 (14.7)	0.081
Private-sector employee	487 (34.3)	330 (36.2)	157 (31)		181 (31.4)	306 (36.4)	
Government-sector employee	750 (52.9)	483 (53)	267 (52.8)		339 (58.8)	411 (48.9)	
Monthly family income							
SAR 10,000 or less	674 (47.5)	478 (52.4)	196 (38.7)	0.000 ***	232 (56)	351 (41.7)	0.000 ***
More than SAR 10,000	744 (52.5)	434 (47.6)	310 (61.3)		254 (44)	490 (58.3)	
Mother’s mean daily consumption of F&Vs	3.48 ± 1.94	2.99 ± 1.55	4.36 ± 2.23	0.000 ***	2.68 ± 1.54	4.03 ± 1.99	0.000 ***
≥7 serving/d	317 (22.4)	789 (86.5)	321 (61.7)	0.000 ***	252 (91)	576 (68.5)	0.000 ***
<7 serving/d	1101 (77.6)	123 (13.5)	194 (38.3)		52 (9)	265 (31.5)	

* *p* ≤ 0.05; *** *p* ≤ 0.0001. SD, standard deviation; F&Vs, fruits and vegetables.

**Table 2:** Parental feeding styles and daily consumption of fruits and vegetables among the children.

Variable	All Participants	Fruits		*p* Value	Vegetables		*p* Value
		<1.5 Pieces/d	≥1.5 Pieces/d		<3 Serving Spoons/d	≥3 Serving Spoons/d	
Instrumental feeding	3.12 ± 1.29	3.18 ± 1.27	3.00 ± 1.33	0.022 *	3.18 ± 1.25	2.87 ± 1.43	0.009 **
Control feeding	3.16 ± 1.30	3.05 ± 1.4	3.22 ± 1.2	0.026 *	3.2 ± 1.29	3.013 ± 1.33	0.017 *
Encouragement feeding	3.69 ± 1.09	3.38 ± 1.02	3.57 ± 0.97	0.031 *	3.61 ± 1.10	3.99 ± 1.01	0.001 **
Emotional feeding	3.45 ± 1.00	3.38 ± 1.02	3.57 ± 1	0.001 **	3.42 ± 1.03	3.45 ± 0.09	0.001 **

* *p* ≤ 0.05; ** *p* ≤ 0.001.

**Table 3:** Association of parental feeding styles and children’s consumption of fruits with demographic factors.

Variable	Parental Feeding Styles OR (95% Confidence Interval)			
	Instrumental Feeding	Emotional Feeding	Encouragement Feeding	Control Feeding
Fruits < 1.5 pieces/d				
Mother’s education level				
Secondary school or lower	Reference category			
University or higher	1.029 (0.970–1.092)	0.997 (0.949–1.046)	1.022 (0.992–1.054)	0.997 (0.967–1.028)
Mother’s employment status				
Unemployed	Reference category			
Employed	1.050 (0.997–1.107)	0.998 (0.957–1.041)	0.952 (0.927–0.977)	1.016 (0.989–1.043) **
Monthly family income				
SAR 10,000 or less	Reference category			
More than SAR 10,000	1.062 (1.009–1.118)	0.986 (0.946–1.028)	0.986 (0.961–1.012)	1.048 (1.021–1.076)
Breastfeeding duration				
Less than 6 months	Reference category			
6 months or more	1.065 (1.011–1.123)	0.959 (0.919–1.001)	0.978 (0.953–1.004)	1.042 (1.015–1.071)
Mother’s mean daily consumption of F&Vs				
<7 serving/d	Reference category			
≥7 serving/d	0.981 (0.908–1.59)	1.115 (1.045–1.190)	0.990 (0.953–1.029)	0.985 (0.948–1.024)
Fruits ≥ 1.5 pieces/d				
Mother’s education level				
Secondary school or lower	Reference category			
University or higher	1.057 (0.970–1.152)	0.936 (0.877–0.999) *	1.067 (1.023–1.112) *	0.921 (0.960–1.046)
Mother’s employment status				
Unemployed	Reference category			
Employed	0.972 (0.911–0.1.037)	1.051 (1.002–1.102)	1.017 (0.984–1.051)	1.045 (1.009–1.081)
Monthly family income				
SAR 10,000 or less	Reference category			
More than SAR 10,000	0.957 (0.895–1.023)	1.095 (1.042–1.51) **	1.049 (1.015–1.085) *	1.009 (0.975–1.046)
Breastfeeding duration				
Less than 6 months	Reference category			
6 months or more	0.941 (0.882–1.004)	1.051 (1.016–1.88) *	0.998 (0.967–1.031)	1.028 (0.981–1.078)
Mother’s mean daily consumption of F&Vs				
<7 serving/d	Reference category			
≥7 serving/d	0.899 (0.841–0.960)	1.049 (0.99–1.012)	1.113 (1.041–1.190) *	1.025 (0.990–1.062)

* *p* ≤ 0.05; ** *p* ≤ 0.001. OR, odds ratio; F&Vs, fruits and vegetables.

**Table 4:** Association of parental feeding styles and children’s consumption of vegetables with demographic factors.

Variable	Parental Feeding Styles OR (95% Confidence Interval)			
	Instrumental Feeding	Emotional Feeding	Encouragement Feeding	Control Feeding
Vegetable < 3 serving spoons/d				
Mother’s education level				
Secondary school or lower	Reference category			
University or higher	1 (0.930–1.075)	0.977 (0.925–1.032)	1.018 (0.982–1.057)	0.986 (0.95–1.024)
Mother’s employment status				
Unemployed	Reference category			
Employed	1.037 (0972–10105)	1.022 (0.974–1.073)	0.963 (0.932–0.995)	1.013 (0.980–1.046) *
Monthly family income				
SAR 10,000 or less	Reference category			
More than SAR 10,000	1.040 (0.977–1.107)	1.001 (0.956–1.049)	0.997 (0.966–1.029)	1.025 (0.993–1.059)
Breastfeeding duration				
Less than 6 months	Reference category			
6 months or more	1.027 (0.965–1.093)	0.999 (0.953–1.046)	1.001 (0970–1.033)	1.007 (0.975–1.039)
Mother’s mean daily consumption of F&Vs				
<7 serving/d	Reference category			
≥7 serving/d	1.006 (0.896–1.130)	1.105 (1.009–1.210) *	0.946 (0.893–1.002)	1.001 (0.944–1.061)
Vegetable ≥ 3 serving spoons/d				
Mother’s education level				
Secondary school or lower	Reference category			
University or higher	1.07 (1.002–1.143) *	0.956 (0.905–1.009)	1.062 (1.029–1.096) **	01.013 (0.980–1.047)
Mother’s employment status				
Unemployed	Reference category			
Employed	0.990 (0.941–1.042)	1.021 (0.980–1.063)	0.988 (0.965–1.031)	1.027 (1.00–1.055)
Monthly family income				
SAR 10,000 or less	Reference category			
More than SAR 10,000	0.891 (0.945–1.050)	1.040 (0.998–1.085)	1.031 (1.006–1.057) *	1.031 (1.003–1.060)
Breastfeeding duration				
Less than 6 months	Reference category			
6 months or more	1.004 (0.951–1.059)	1.070 (1.039–1.101) **	0.971 (0.947–0.997)	0.981 (0.940–1.024)
Mother’s mean daily consumption of F&Vs				
<7 serving/d	Reference category			
≥7 serving/d	0.915 (0.865–0.967)	1.040 (0.995–1.089)	1.049 (1.022–1.077) **	0675 (0.994–1.023)

* *p* ≤ 0.05; ** *p* ≤ 0.001. OR, odds ratio; F&Vs, fruits and vegetables.

## Data Availability

The datasets used and analyzed in the current study are available from the corresponding authors upon reasonable request.
